# A survey of food-borne and antimicrobial resistance-harbouring bacteria in meat by-products from knackeries and associated equipment and kennels

**DOI:** 10.1186/s13620-022-00219-4

**Published:** 2022-05-10

**Authors:** Shannon McDonnell, Montserrat Gutierrez, Finola C. Leonard, Tony O’Brien, Pat Kearney, Catherine Swan, Gillian Madigan, Elaine Bracken, Joanne McLernon, Margaret Griffin, Ciaran M. O’Sullivan, John Egan, Deirdre M. Prendergast

**Affiliations:** 1 Department of Agriculture, Food and the Marine, Backweston Complex, Celbridge, Co., Kildare, Ireland; 2 School of Veterinary Medicine, Veterinary Science Centre, University College Dublin, Belfield, Dublin 4, Ireland

**Keywords:** Meat by-products, Knackery, Antimicrobial resistance, Foodborne zoonotic bacteria

## Abstract

**Background:**

In Ireland, meat by-products (MBP) harvested at knackeries from farmed animals that have not died of an infectious or systemic disease are legally permitted to be fed to dogs in kennels and packs of hounds. There is limited information available on the risks of spreading foodborne bacteria or antimicrobial resistant (AMR) determinants to dogs, their handlers or the associated environment. The aim of this study was to investigate the distribution of *Salmonella* serovars, *Listeria monocytogenes*, *Campylobacter* species*,* enterococci*,* their associated AMR determinants and the level of *Escherichia coli* in samples of MBP from knackeries and associated equipment and kennels. For this purpose, 313 fresh and 208 frozen MBP samples from 22 knackeries, 16 swabs of mincing equipment from two of the knackeries and 138 swabs from kennels adjacent to seven of the knackeries were collected and processed over a 12-month period.

**Results:**

From the 521 MBP samples analysed, a total of 77 *Salmonella* (14.8%), 101 *L. monocytogenes* (19.4%), 12 *Campylobacter* (2.3%), 271 *Enterococcus faecalis* (52.0%) and 127 *Enterococcus faecium* (24.4%) strains were recovered. The 154 analysed environmental samples from kennels and mincing equipment yielded 194 isolates (3 *Salmonella*, 85 *E. coli*, 76 *E. faecalis* and 30 *E. faecium*.). *E. coli* was quantifiable in 423 of the 521 MBP samples with log counts per gram ranging between 1 and 6. AMR characterisation of 168 *E. coli*, enterococci and *Salmonella* isolates from MBP and environmental samples showed high levels of AMR including multi-drug resistance (MDR) with 63.6%, 9.1%, 29% and 45.8% of *E. coli*, *Salmonella*, *E. faecalis* and *E. faecium* isolates, respectively showing resistance to three or more antimicrobials (MDR)

**Conclusions:**

The findings of this survey confirm that MBP from fallen animals contain high levels of zoonotic and AMR-harbouring bacteria that pose a risk of transmission to dogs, their handlers, and the environment.

## Introduction

In Ireland, when an animal dies on a farm as a result of injury or a non-notifiable disease, the carcass must be transported to a rendering plant or to a knackery for processing and disposal. Meat by-products (MBP) harvested at knackeries from farmed animals that have not died of an infectious or systemic disease are legally permitted to be fed to dogs in kennels and packs of hounds. During 2020 there were 228,257 recorded on-farm cattle deaths (excluding stillborn) in Ireland [1].

To prevent risks to public and animal health and to ensure that fallen animals do not enter the food chain, knackeries must adhere to strict rules regarding the collection, transport, storage, use, processing and disposal of MBP. Regulation (EC) No. 1069/2009 [2] permits the feeding of dogs from listed kennels and packs of hounds and dogs in shelters with MBP harvested at approved knackeries from low risk fallen farmed animals. While greyhounds and hunting dogs are currently classified as farm animals under Irish law [3], dogs in general are recognised as carriers of zoonotic bacteria, showing no clinical signs of disease in many cases [4, 5], but there are also some descriptions of fatal salmonellosis and campylobacteriosis in dogs as a consequence of contaminated pet foods [6, 7].

In recent years, raw pet food diets have become popular for dogs and are considered both nutritious and healthy [6, 8]. While the benefits of feeding dogs with a raw meat diet have been previously claimed [6, 8], other studies have outlined potential risks with these diets, i.e., bacterial infections, parasitic disease and nutrient imbalance [9–12]. Foodborne zoonotic bacteria such as *Salmonella*, *Listeria monocytogenes* and *Campylobacter* have all been identified in raw pet foods [13–15] and disease associated with feeding *Salmonella*-contaminated raw feeds to greyhounds has been previously reported [16].

Data relating to bacterial contamination of MBP used as feed for kennel dogs is scarce, particularly in relation to Ireland and there is currently a lack of information available on the risks of spreading foodborne bacteria or AMR determinants to dogs, their handlers or the environment which may be associated with the practice of feeding MBP from fallen animals to dogs. Therefore, the aim of this survey was to investigate the presence of food-borne and antimicrobial resistance-harbouring bacteria in MBP, mincing equipment and kennels where MBP are fed to dogs to assess the risks associated with this practice in Ireland.

## Materials and Methods

### Knackery selection and sampling

For this survey, we selected 22 of the 37 registered knackeries in the Republic of Ireland based on 1) nationwide distribution, 2) approval for harvesting MBP and 3) willingness to take part in the survey. From each premises, fresh and frozen (if available) bovine MBP 500 g samples were collected using sterile kits at regular intervals from January to December 2016 by a Department of Agriculture, Food and the Marine (DAFM) official veterinarian. A total of 313 fresh and 208 frozen MBP samples were obtained. Information on herd type, age, recent drug treatment or the health status of dogs was not available.

Mincing equipment for processing MBP was on site in two of the knackeries and, for those premises, swabs were obtained by swabbing the entire inside surface and head of the mincer using a sterile sponge (Helapet, Bedfordshire, UK). A total of 16 mincing equipment swabs were collected.

Kennels adjacent to seven knackeries were swabbed at quarterly intervals over the 12-month period. Kennels varied in size (from 10 to 100 dogs) and breed types (mainly foxhounds and greyhounds although one had numerous breeds). Six locations had kennels made from concrete (walls and floors) and kennel sizes varied from 8 x 8 ft to 12 x 14 ft. The smallest had 2-4 random breeds per kennel and the largest was a hunt kennel which housed approximately 30-40 hounds per kennel. The seventh location had random small kennels scattered around the knackery/farm and these kennels had concrete floors with wired fencing. For the majority of visits the dogs were not in the kennels when sampling took place and kennels appeared to have been hosed down prior to sampling. On occasions where cleaning had not been conducted, bedding with faeces was also collected. Five different areas within each kennel were randomly sampled, taking precautions to avoid cross contamination.

Using disposable gloves, a sterile pre-moistened sponge (Helapet, Bedfordshire, UK) was used to sample each of the five areas by dragging across a 0.24 m^2^ area and then returning the sponge to its sterile bag. Faecal samples, n=21, were also collected. In total 138 kennel swabs including faecal samples were collected. All samples were placed in a cool box containing ice blocks and transported to the laboratory within 6 h. Once received at the laboratory, fresh samples and environmental samples were tested within 24 h while frozen MBP samples were stored at -20°C pending testing.

### Sample preparation and microbiological examination

MBP samples were processed for the detection of *Salmonella*, *L. monocytogenes, Campylobacter* (in 25 g), *Enterococcus* and *E. coli* (in 1g) and for the enumeration of *E. coli*. Environmental samples (i.e., equipment and kennel samples and faecal samples) were examined for the presence of *Salmonella*, *Enterococcus* and *E. coli*.

Detection of *Salmonella* was based on ISO 6579-1:2017 [17]. For each bacterial species, one suspect colony per plate was selected for confirmation. Detection of *L. monocytogenes* was based on ISO 11290-1:1996 [18]. Detection of thermotolerant *Campylobacter* spp. was based on ISO 10272-1:2006 [19] with modifications to include Bolton broth as the initial enrichment medium. Enumeration of *E. coli* was performed according to ISO 16649-2:2001 [20]. Detection of *Enterococcus* was based on Wegener et al. (1997) [21] and Ahmad et al. (2002) [22] modified to include the addition of one gram of MBP to 9 ml of Brain Heart Infusion broth (BHI; Sigma, Missouri, United States) supplemented with 6% Sodium Chloride and subsequent subculture on Slanetz-Bartley agar (E&O, Bonnybridge, Scotland). Detection of *E. coli* was carried out according to Tanih et al. (2015) [23] with some minor modifications.

All isolates were identified using MALDI-ToF (Matrix Assisted Laser Desorption Ionization-Time of Flight) Mass Spectrometry (Bruker Daltronics GmbH, Bremen, Germany) as described by Ramovic et al. (2020) [24]. MALDI-ToF also permitted speciation of enterococci and *Campylobacter*. *Salmonella* isolates were typed according to the Kauffman-White-Le Minor scheme, using somatic (O) and flagellar (H) antigens (Sifin Diagnostics, Berlin, Germany) as described by Prendergast et al. (2012) [25] and if necessary using Polymerase Chain Reaction (PCR) for identification of Monophasic *S*. Typhimurium according to Prendergast et al. 2013 [26].

### Antimicrobial Susceptibility Testing

The antibiotic susceptibility profiles of the isolates were determined using the mandatory harmonised method that is outlined in the EU Commission Implementing Decision 2013/652/EU [27]. The epidemiological cut-off values and the concentration ranges that are set out in Tables 1, 2 and 3 of the decision were used.

**Table 1:** Bacterial pathogens detected in fresh and frozen MBP samples

Pathogen	No. (%) positive		
	Fresh (***n*** = 313)	Frozen (***n*** = 208)	Total (***n*** = 521)
*L. monocytogenes*	59 (18.8%)	42 (20%)	101 (19.4%)
*Salmonella* spp.	52 (16.6%)	25 (12%)	77 (14.8%)
*Campylobacter* spp.	10 (3.2%)	2 (1%)	12 (2.3%)
*Enterococcus faecalis*	158 (50.5%)	113 (54%)	271 (52%)*
*Enterococcus faecium*	87 (27.8%)	40 (19%)	127 (24%)*

**Table 2:** Detection of *Salmonella* spp., *E. coli*, *E. faecalis* and *E. faecium* from environmental samples

	No. (%) positive		
	Kennel (***n***=138)	Mincing (***n***=16)	Total (***n***=154)
*Salmonella*	0 (0%)	3 (18.8%)	3 (1.9%)
*E. coli*	77 (55.8%)	8 (50%)	85 (55.3%)
*E. faecalis*	67 (48.6%)	9 (56.3%)	76 (49.4%)
*E. faecium*	28 (20.3%)	2 (12.5%)	30 (19.5%)

**Table 3:** Antimicrobial resistance of *E. coli* isolates recovered from MBP (N=37) and environmental samples (N=40)

Antimicrobial*	MBP (%)	Environmental samples (%)
Ampicillin	21 (56.8)	21 (52.5)
Cefotaxime	2 (5.4)**	1 (2.5)***
Ceftazidime	2 (5.4)**	0
Chloramphenicol	19 (51.4)	18 (45)
Ciprofloxacin	16 (43.2)	16 (40)
Gentamicin	9 (24.3)	2 (5)
Nalidixic acid	16 (43.2)	14 (35)
Sulfamethoxazole	28 (75.5)	23 (57.5)
Tetracycline	28 (75.7)	23 (57.5)
Trimethoprim	22 (59.5)	13 (32.5)
Fully susceptible	9 (24.3)	15 (37.5)

A total of 168 isolates were selected for antimicrobial susceptibility, including 37 *E. coli* and 30 *Salmonella* from MBP, 40 *E. coli* (2 mincer, 31 kennels, 7 faeces), 3 *Salmonella* (mincer), 34 *E. faecalis* and 24 *E. faecium* (9 mincer, 39 kennels and 10 faeces) from environmental samples. Selection aimed to generate maximum information within the constraints of economic and time limitations.

Antimicrobial susceptibility testing was carried out as described by Ramovic et al. (2020) [24]. Minimum Inhibitory Concentration (MIC) for relevant antimicrobials was determined in broth microdilution antimicrobial susceptibility assays using different commercially available Sensititre plates (Thermo Fisher Scientific, Massachusetts, United States). *E. coli* and *Salmonella* isolates were tested initially using the EUVSEC plate and strains which exhibited resistance to cefotaxime, ceftazidime and/or meropenem were considered suspect extended spectrum beta-lactamase (ESBL) producers and further tested with the EUVSEC2 plate. *Enterococcus* strains were tested using the EUVENC plates. Suitable controls strains, *E. coli* 25922 and *E. faecalis* 29212, were tested with each batch of samples and the classification of the phenotypic results was based on EU Commission Decision 2013/652/EU [27] and the most recent EFSA recommendations as described by Ramovic et al. (2020) [24].

Isolates were deemed MDR when resistance was found to three or more antimicrobials.

## Results

### MBP Samples

A total of 190 strains of potentially zoonotic bacteria were isolated from MBP as shown in Table 1. *L. monocytogenes* was the most frequently isolated bacterium of the three classical foodborne pathogens, both in fresh and frozen samples.

Among the 77 *Salmonella* isolates, a total of 7 serovars were identified. *S*. Dublin was the most frequently isolated (*n*=39) followed by *S*. Typhimurium (*n*=8), *S*. Montevideo (*n*=8), Monophasic *S*. Typhimurium (*n*=5), *S*. Braenderup (*n*=4), *S*. Anatum (*n*=2) and *S*. Agama

(*n*=1). Ten *Salmonella* isolates’ antigenic formula could not be fully ascertained by serotyping and were therefore designated as *S.* Unnamed.

*Campylobacter* in MBP was isolated in 10 fresh and 2 frozen samples. MALDI-ToF identified the isolates as *C. fetus* subsp. *intestinalis* (*n*= 6)*, C. coli* (*n*=4) and *C. jejuni* (*n*=2). C*. fetus* subsp. *intestinalis* was found in fresh samples only.

**Fig. 1:**
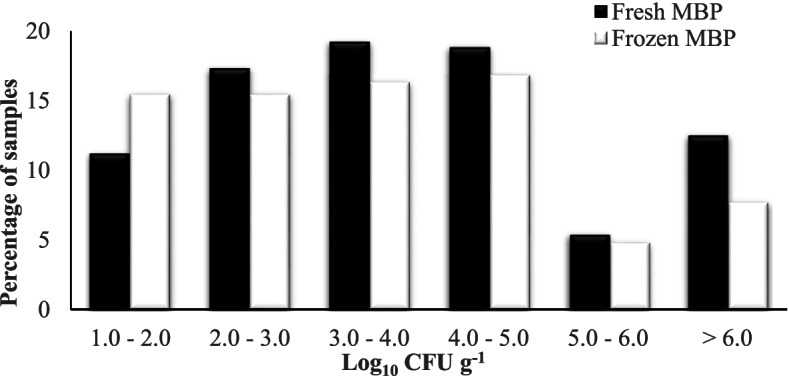
Summary of *E. coli* counts recovered from fresh and frozen MBP samples

The recovery rate of *E. faecalis* from MBP was greater than that of *E. faecium* for both fresh and frozen samples (Table 1). *E. coli* was recovered from 423 of 521 (81%) of MBP samples at varied levels in fresh and frozen samples as shown in Fig. 1. The number of *E. coli* recovered from fresh MBP was generally numerically greater than from frozen MBP.

### Environmental samples

A total of 3 *Salmonella,* 85 *E. coli,* 76 *E. faecalis* and 30 *E. faecium* isolates were recovered from environmental samples as shown in Table 2. *E. coli* was isolated from 77 out of 138 kennel samples and 8 out of 16 mincing equipment samples (55.8% and 50% respectively). *S.* Braenderup*, S.* Dublin and *S*. Typhimurium, one of each, were isolated from mincing equipment (3 out of 16, i.e., 18.8%) but not from kennels, while *Enterococcus* were recovered from kennel samples (67 *E. faecalis* and 28 *E. faecium* out of 138 samples or 48.6% and 20.3% respectively), and mincing equipment (9 *E. faecalis* and 2 *E. faecium* out of 16 samples or 56.3% and 12.5% respectively).

### Antimicrobial Resistance

Broth microdilution AMR susceptibility testing results from the MBP and environmental isolates are shown in Tables 3, 4 and 5. Overall, isolates ranged from fully susceptible to resistant to 10 antimicrobials with a large proportion of isolates resistant to more than three antimicrobials. AMR testing on *E. coli* isolates recovered from MBP showed a high level of resistance to tetracycline, sulfamethoxazole, trimethoprim, ampicillin, chloramphenicol, ciprofloxacin and nalidixic acid in both MBP and environmental samples with 76% of MBP and 58% of Environmental isolates resistant to tetracycline and sulfamethoxazole. Based on the results obtained in the EUVSEC2 plate, two isolates were identified as presumptive plasmid mediated AmpC (pAmpC) and one as presumptive ESBL producing *E. coli* (Table 3). The two presumptive pAmpC producing *E. coli* were resistant to seven and nine antimicrobials and the presumptive ESBL positive isolate recovered from the environmental kennel swab was resistant to nine antimicrobials.

**Table 4:** AMR profiles of *Salmonella* isolates from MBP and environmental samples

Antimicrobial resistance profile*	Serotype	MBP	Environmental samples
		**(n=30)**	**(n=3)**
Fully susceptible	*S*. Dublin	16	1
Fully susceptible	*S.* Braenderup	3	1
Fully susceptible	*S.* Typhimurium	2	1
Fully susceptible	*S*. Agama	1	-
Fully susceptible	*S.* Montevideo	1	-
Fully susceptible	Unnamed	2	-
AMP TET	monophasic *S*. Typhimurium	1	-
CIP NAL	*S*. Dublin	1	
AMP CHL TET	*S*. Typhimurium	3	-

**Table 5:** Antimicrobial resistance of *E. faecalis* (*N*=34) and *E. faecium* (*N*=24) from environmental samples

Antimicrobial*	Environmental samples (n= 40)	
	***E. faecalis*** (%)	***E. faecium*** (%)
Ampicillin	0	2 (8.3)
Chloramphenicol	12 (35.3)	4 (16.7)
Ciprofloxacin	0	4 (16.7)
Daptomycin	1 (2.9)	10 (41.7)
Erythromycin	11 (32.4)	7 (29.2)
Gentamicin	2 (5.9)	0
Linezolid	3 (8.8)	1 (4.2)
Quinupristin/Dalfopristin	NA	11 (45.8)
Tetracycline	27 (79.4)	17 (70.8)
Fully susceptible	5 (14.7)	2 (8.3)

The monophasic *S.* Typhimurium isolated from frozen MBP was resistant to both ampicillin and tetracycline (Table 4). Amongst the six *S*. Typhimurium isolates, three were fully susceptible and three were resistant to three antimicrobials. The majority of the *S*. Dublin isolates were fully susceptible, and one *S.* Dublin isolate from a frozen MBP sample was resistant to both ciprofloxacin and nalidixic acid.

Among the 34 *E. faecalis* and the 24 *E. faecium* isolates examined for AMR, tetracycline resistance was the most common (79.4% of *E. faecalis* and 70.8% of *E. faecium*) followed by chloramphenicol and erythromycin in *E. faecalis* and quinupristin/dalfopristin, daptomycin, chloramphenicol and ciprofloxacin in *E. faecium* (Table 5).

## Discussion

In Ireland it is common practice to feed MBP from knackeries to greyhounds and foxhounds as allowed under SI 252/2008 [28], and this MBP is most often fed raw. The controls governing the feeding of MBP recovered at knackeries from fallen animals to associated kennels as per EU Regulation (EC) 142/2011 [29] are much less stringent than those governing meat destined for human consumption and there are no previous studies evaluating the risks associated with such products in Ireland.

The results of our study indicate that MBP from fallen animals contain potential pathogens including *Salmonella* and *Campylobacter*. As *Salmonella* may be carried in the gastrointestinal tract of ruminants it was not surprising that 14.8% of MBP samples were contaminated with this pathogen. The lower recovery rate of *Salmonella* in frozen samples when compared to fresh may be explained by lower viability after freezing as has been observed by other authors [30].

Reported prevalence of *Salmonella* in bones and raw pet foods in previous studies varied greatly, from 0.2% [31], 5.9% [32], 12% [33], 20% [10], to 80% [34].

Although 14.8% of MBP contained *Salmonella*, *Salmonella* spp. was not isolated from kennels. This finding differed from published data but could be explained by the type of MBP and the limited number of kennels sampled. *S.* Dublin which was the most prevalent serovar from MBP is host adapted to cattle and may not persist in the gastrointestinal tract of dogs to the same extent as other serotypes; *S.* Dublin accounted for only 9% of *Salmonella* isolated from dogs in the UK from 1954 – 2012 [35].

*L. monocytogenes* is a ubiquitous organism commonly found on dairy and beef farms. A study conducted by Fox et al. (2009) [36] investigated the prevalence of *L. monocytogenes* on 16 Irish farms. Of 298 environmental samples collected, 19% of samples were positive for *L. monocytogenes* indicating its widespread distribution in cattle farms. Overall, this organism was the most frequently isolated pathogen in fresh (18.8%) and frozen (20.2%) samples. A clear link between cleanliness and contamination with *L. monocytogenes* has been established [36, 37]. As knackeries do not undertake the hygiene practices found in establishments producing meat for human consumption it is therefore not unexpected to find these contamination levels. Even higher isolation rates have been described, with 54% *L. monocytogenes* isolation rate in frozen raw pet food reported by Van Bree et al. (2018) [10].

A low prevalence of *Campylobacter* was found in this study, in agreement with published literature [38–40], although Bojanić et al. (2017) [41] reported a high prevalence of *Campylobacter* in raw dog food. *C*. *fetus* subsp *intestinalis* which was predominantly recovered from fresh samples, naturally resides in the gastrointestinal tract of cattle and has seldom been linked to human disease [42], while *C. coli* and *C. jejuni* are recognised pathogens of humans and are common commensals of cattle, sheep, pigs, and other species of domestic and wild animals [43]. Although the occurrence of *Campylobacter* in MBP in this study was low, there is still a risk of transmission to dogs [8, 44] and dogs have been reported to shed this organism after consumption of raw meat [15].

Sterilisation, heat processing or freezing prior to feeding has been reported to reduce the bacterial load [45]. In this study, fewer *Salmonella*, *Campylobacter* and *E. coli* were found in frozen MBP than in fresh MBP, although freezing had little impact on levels of *L. monocytogenes* and enterococci. Samples analysed for *E. coli* and enterococci showed high rates of contamination, reflecting the low standard of hygiene practices in knackeries. The level of *E. coli* was 4 log or greater in approximately 35% of MBP samples, thus exceeding the absolute threshold of 5,000 CFU/g stipulated in Commission Regulation (EU) No 142/2011 for raw pet food at the point of production [29]. Although this regulation does not apply to the feeding of dogs with MBP supplied directly from knackeries on site, our findings demonstrate the high level of microbial contamination in MBP.

Apart from the risks posed by the presence of zoonotic organisms in MBP, contamination with antimicrobial resistant bacteria also constitutes a potential risk for human and animal health. In recent years the role of companion animals in transmission of AMR has been investigated by several authors including Damborg et al. (2016) [5] who identified dogs as a source of infection of antimicrobial resistant bacteria. High levels of AMR to ampicillin, ciprofloxacin, chloramphenicol and tetracycline were found in both *E. coli* and enterococci isolates from kennels. In addition, resistance to nalidixic acid, trimethoprim and sulphamethoxazole was high in *E. coli* and resistance to daptomycin and quinupristin/dalfopristin in *E. faecium*. The patterns of AMR were similar among *E. coli* isolates recovered from both environmental and MBP samples. The high level of resistance to tetracyclines, sulphonamides, trimethoprim and ampicillin seen in MBP and environmental isolates of *E. coli* is likely the result of selective pressure as a result of antimicrobial treatment of fallen stock and/or more frequent use of these antimicrobials within veterinary medicine, resulting in *E. coli* strains colonising the bovine gastrointestinal tract and subsequent transmission of these resistant organisms to dogs after feeding MBP [46].

Three ESBL producing *E. coli* ss detected in this study. ESBL producing *E. coli* have been isolated from numerous different animal sources, including dogs. A study performed by Baede et al. (2015) [47], suggested a correlation between feeding dogs a raw diet that contains ESBL-producers and the presence of these *E. coli* in dogs. In this work, one ESBL isolate was identified in a kennel environmental sample.

Additional labelling on MBP samples to declare the presence of harmful bacteria and recommendations on handling MBP is advisable to make kennel operators more aware of the potential risks of MBP. The possible development of a standard operating procedure for harmonisation of sanitation practices across all knackeries should be considered. Comprehensive cleaning procedures to include all equipment and all surfaces before and after processing should be implemented. In addition, measures such as effective hand washing, general hygiene and personal protective equipment should be included in protocols as effective prevention measures against zoonotic infections.

## Conclusion

This is the first study to document the risks associated with feeding MBP from fallen animals to dogs. The findings indicate that MBP may be a vehicle for transmission of zoonotic pathogens and antimicrobial resistant determinants to dogs, their owners and the environment. Our findings may serve as the focus for future research to understand the risks to human and animal health associated with feeding this type of product to dogs.

As MBP is a rich nutrient matrix which supports the growth of bacteria including pathogens, reducing to zero the microbial load and all risks for product handlers, dogs and their owners is not achievable and enhanced control measures should be considered instead.
